# Cardiac arrest as the first presentation of Takayasu arteritis in a young adult: a case report

**DOI:** 10.1093/ehjcr/ytag207

**Published:** 2026-03-18

**Authors:** Débora da S Correia, Marisa Trabulo, Sância Ramos, Tiago Nolasco, António Tralhão

**Affiliations:** Cardiology Department, Hospital de Santa Cruz, ULSLO, Av. Prof. Dr. Reinaldo dos Santos, Carnaxide, Lisbon 2790-134, Portugal; Cardiology Department, Hospital de Santa Cruz, ULSLO, Av. Prof. Dr. Reinaldo dos Santos, Carnaxide, Lisbon 2790-134, Portugal; Pathology Department, Hospital de Santa Cruz, ULSLO, Av. Prof. Dr. Reinaldo dos Santos, Carnaxide, Lisbon 2790-134, Portugal; Cardiac Surgery Department, Hospital de Santa Cruz, ULSLO, Av. Prof. Dr. Reinaldo dos Santos, Carnaxide, Lisbon 2790-134, Portugal; Intensive Care Unit, ECMO Reference Centre, ULSSJ, Rua José António Serrano, Lisbon 1150-199, Portugal

**Keywords:** Out-of-hospital cardiac arrest, Takayasu, Coronary artery disease, Vasculitis, Case report

## Abstract

**Background:**

Sudden cardiac arrest (SCA) is rare in young individuals, being typically caused by inherited cardiomyopathies or electrical disorders. However, less common aetiologies should not be overlooked.

**Case summary:**

A male in his twenties suffered an aborted SCA. Initial investigations revealed significant triple-vessel coronary artery disease leading to urgent surgical revascularization. Macroscopically, diffuse coronary luminal narrowing due to marked wall thickening was observed. Histopathology of the right internal mammary artery showed a non-necrotizing vasculitis pattern, which, together with abdominal aorta, mesenteric and iliac artery involvement on computed tomography angiography, supported a diagnosis of Takayasu arteritis. High-dose glucocorticoids were started, resulting in rapid normalization of inflammatory biomarkers. The patient was discharged after 54 days, with programmed administration of anti-tumour necrosis factor-alpha monoclonal antibody adalimumab.

**Discussion:**

Takayasu arteritis is a rare vasculitis classically affecting medium- to large-sized vessels, but may involve the coronary tree and ultimately manifest as SCA. This case underscores the diagnostic challenges of young adults presenting with acute coronary syndromes, highlighting the importance of clinical suspicion, histopathological evaluation, and a collaborative approach for a successful outcome.

Learning points

- Although rare, vasculitis should be considered as a possible aetiology of coronary artery disease in young adults with no atherosclerotic risk factors.
- Sudden cardiac arrest can be the first manifestation of Takayasu arteritis.
- Extensive investigation including histopathology, coupled with interdisciplinary collaboration, is essential to a final diagnosis and adequate treatment initiation.

## Introduction

Takayasu arteritis (TA), is a rare, chronic, large- to medium-sized vasculitis that predominantly affects the aorta and supra-aortic trunks. Coronary artery involvement has been described in 10%–30% of cases.^1,2^ The estimated incidence is 2.6 per million individuals per year, with a peak onset in the second and third decades of life.^3^ Clinical presentation depends on which territories are involved, ranging from asymptomatic with an incidental diagnosis to catastrophic cases. The occurrence of sudden death is rare, with only a few cases being previously reported.^4–7^

## Summary figure

**Figure:**
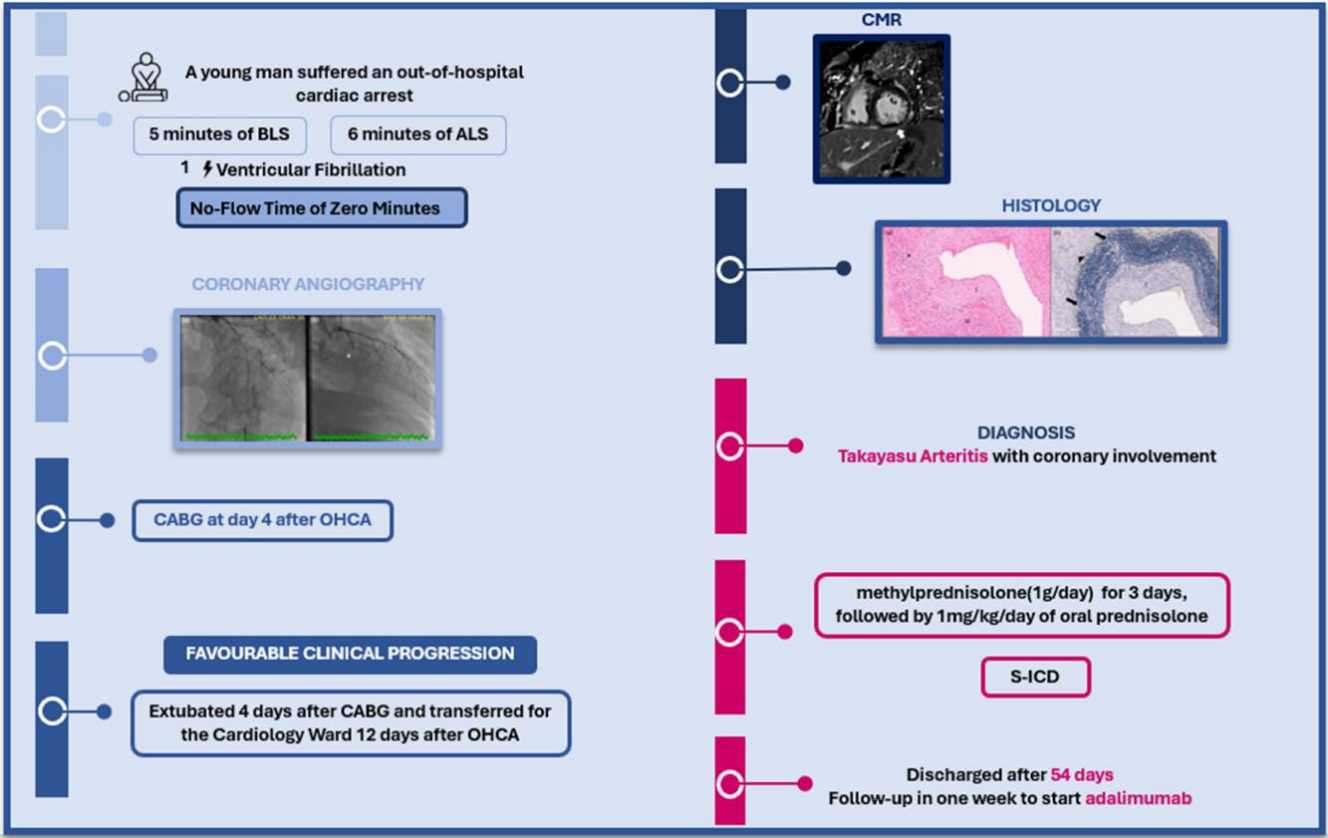
After experiencing an out-of-hospital cardiac arrest, a young man underwent coronary angiography, which revealed (a) severe diffuse luminal narrowing and (b) occlusion of a large obtuse marginal (OM) artery (*asterisk*). Surgical revascularization was performed 4 days after the arrest, followed by a favourable clinical course that allowed for extubation and transfer to the Cardiology Ward. Cardiac magnetic resonance imaging (CMR) demonstrated ischaemic scarring (*white arrow*). Histological analysis was crucial for the final diagnosis of TA. (a) Elastic artery with intimal thickening (I) and medial layer (M) damage due to inflammatory infiltrate (*asterisk*) (H&E). (b) Elastic stain with intimal thickening (I), marked disruption of elastic fibres in the medial layer (*arrows*), and fibrous thickening of adjacent tissue (*arrowhead*) (elastic van Gieson). He was started on glucocorticoids and implanted an S-ICD, being discharged after 54 days of hospitalization with a follow-up in 1 week to start adalimumab. BLS, Basic Life Support; ALS, Advance Life Support; OM, obtuse marginal artery; OHCA, out of hospital cardiac arrest; CABG, coronary artery bypass grafting; CMR, cardiac magnetic resonance imaging; TA, Takayasu arteritis; H&E, haematoxylin–eosin; S-ICD, subcutaneous implantable cardioverter-defibrillator.

## Case presentation

A healthy male patient in his late twenties with no substance abuse or relevant family history suffered an out-of-hospital cardiac arrest after a run on the beach. Return of spontaneous circulation occurred after 5 min of basic, followed by 6 min of advanced life support (no-flow time of 0 min), during which he received one defibrillator shock for ventricular fibrillation, and mechanical ventilation. Twelve-lead electrocardiogram (ECG) showed sinus rhythm with widespread ST-segment depression in leads V2-V6, aVL, I and II, and ST elevation in aVR. Emergent coronary angiography was deferred due to low likelihood of occlusive myocardial infarction. He was admitted to a polyvalent intensive care unit for further evaluation.

Initial physical examination was unremarkable. Repeated 12-lead ECG showed normalization of ST-segment. Maximum high-sensitivity troponin I reached 51 825 ng/L, with no other significant laboratory abnormalities. A transthoracic echocardiogram revealed a non-dilated, non-hypertrophied left ventricle, with moderate impairment of left ventricular function (ejection fraction of 35%), hypokinesia of the distal half of the interventricular septum, inferior wall, and distal thirds of the lateral and anterior walls. Assuming an acute coronary syndrome, he was referred for coronary angiography within 24 h of cardiac arrest.

**Figure 1:**
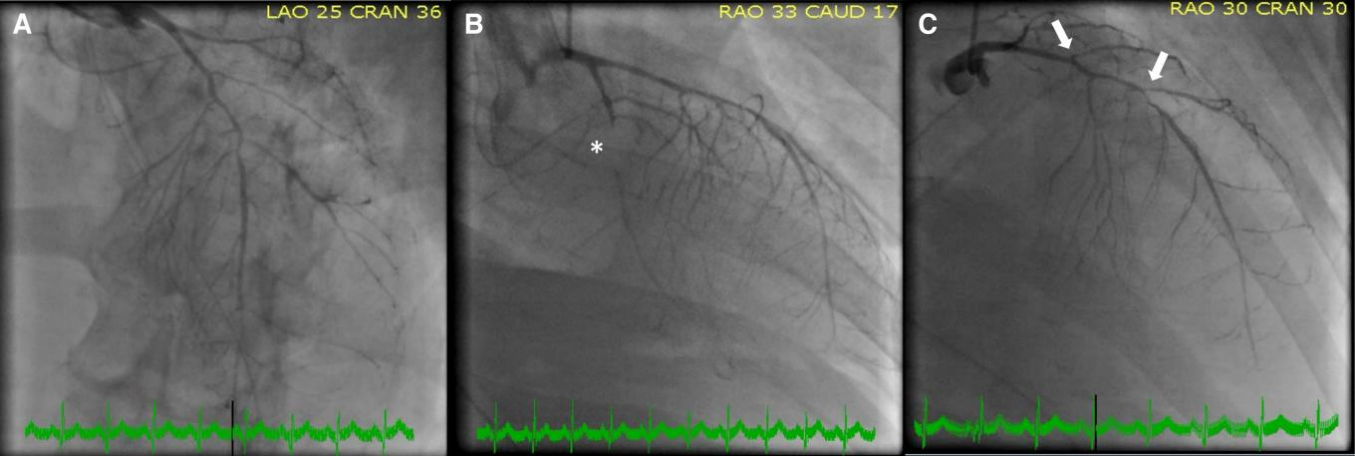
Coronary angiography. Coronary angiography showing (*A*) severe diffuse luminal narrowing; (*B*) occlusion of a large OM (asterisk); and (*C*) LAD presenting mid-segment narrowing and stenosis at the origin of the second and third diagonal branches (arrows). OM, obtuse marginal artery; LAD, left anterior descending artery.

Coronary angiography showed triple-vessel disease with severe diffuse luminal narrowing. The left main artery was free of lesions; the left anterior descending artery (LAD) exhibited a long mid-segment narrowing and apical occlusion. There was stenosis at the origin of the second and third diagonal branches and occlusion of a large obtuse marginal artery (OM). The right coronary artery (RCA) was diffusely irregular (*Figure 1*; Supplementary material online, *Video S1*). Coronary artery bypass grafting (CABG) was performed 3 days later. Intraoperatively, coronary walls appeared thickened. Substantial retrosternal vascular plexus adhesions required laborious arterial dissection. The proximal two-thirds of the left internal mammary artery (LIMA) were harvested. The right internal mammary artery (RIMA) was only removable *en bloc* with the entire vascular plexus and fascia, revealing intimal dissection throughout, leading to conduit abandonment and bilateral saphenous vein harvesting. Triple bypass was performed without extracorporeal circulation: a LIMA graft to the LAD, a saphenous vein graft (with proximal ‘T’ anastomosis to the LIMA) to the second OM and a final saphenous graft to the posterior descending artery. A segment of the RIMA was sent for histology.

He showed favourable progression, enabling successful sedation weaning and extubation four days after surgery.

Laboratory tests showed a normal metabolic profile. Serum C-reactive protein (CRP) and erythrocyte sedimentation rate (ESR) were 16 mg/dL and 88 mm/h, respectively. All serologies, including anti-Treponema antibody, were negative. Immunological testing was negative for anti-nuclear antibodies, extractable nuclear antigen antibodies, cryoglobulins, and IgG4-related disease.

**Figure 2:**
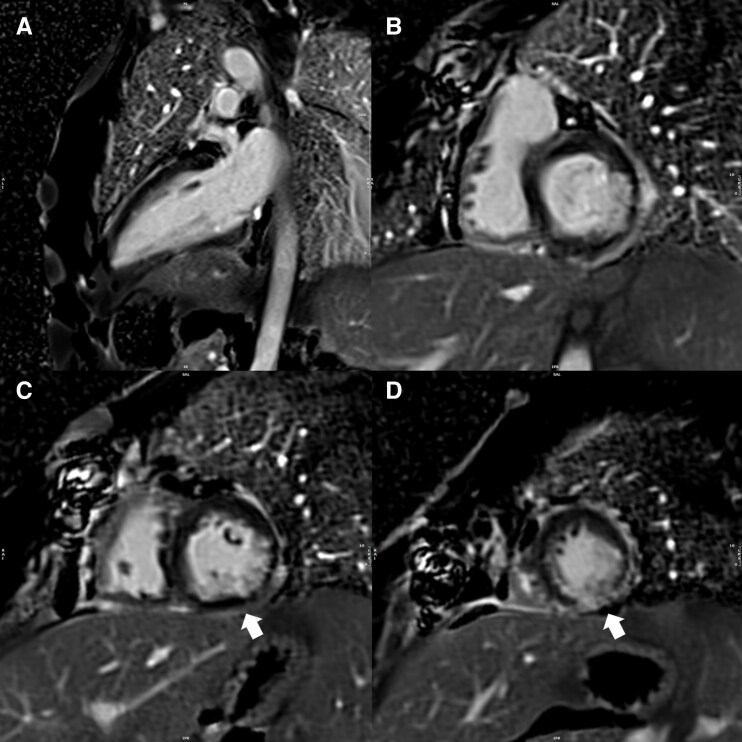
CMR. Cardiac MRI revealing ischaemic scarring. (*A*) Two-chamber view demonstrating subendocardial LGE with 75% of transmurality in the inferior wall. (*B–D*) Basal to apical SAX view also highlighting LGE in the inferolateral and inferior walls (arrows). SAX, short-axis; LGE, late gadolinium enhancement.

Computed tomography (CT) angiography revealed lumen narrowing in the infrarenal abdominal aorta and common iliac arteries, with changes in the inferior mesenteric artery and mild irregularities in the hepatic and superior mesenteric arteries. Cardiac MRI (CMR) showed mild left ventricular dysfunction (ejection fraction of 44%) and ischaemic scarring in the inferolateral and inferior walls (*Figure 2*).

**Figure 3:**
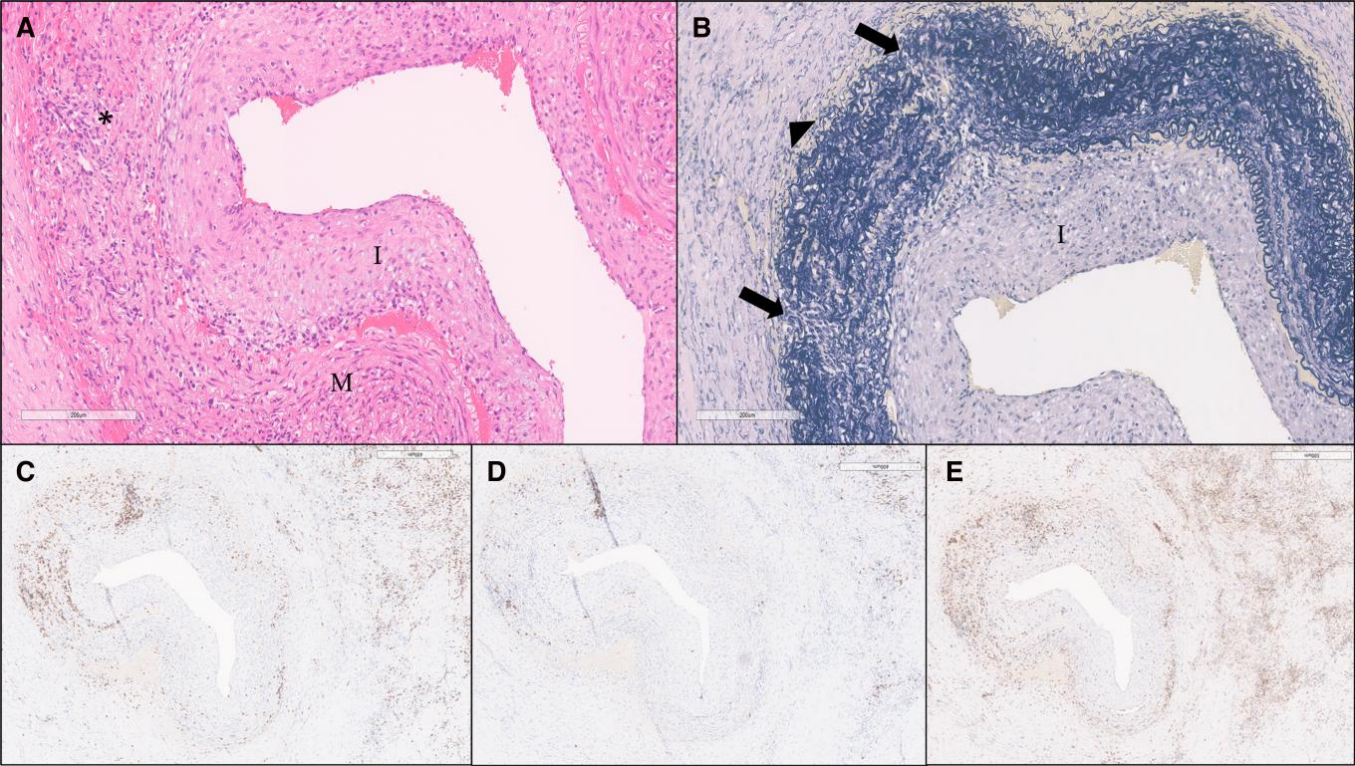
Histological analysis with immunohistochemistry of patient’s RIMA. A segment of the RIMA was sent for histology and it was crucial to final diagnosis. (*A*) Elastic artery (RIMA) with intimal thickening (*I*) and medial layer (*M*) damage due to inflammatory infiltrate (asterisk) (H&E). (*B*) Elastic stain with intimal thickening (*I*), marked disruption of elastic fibres in the medial layer (arrows), and fibrous thickening of adjacent tissue (arrowhead) (elastic van Gieson). (*C–E*) Immunohistochemistry: CD3+ (T lymphocytes) and CD20+ and CD68+ (histiocytic cells), respectively. This highlights the inflammatory infiltrates corresponding to the areas of medial disruption seen in (*B*) and the inflammatory infiltrates identified in (*A*), also present in the adjacent tissue. RIMA, right internal mammary artery; H&E, haematoxylin–eosin.

Histopathological examination of the RIMA showed disruption in the elastic fibres of the tunica media and vessel wall infiltration by inflammatory cells predominantly composed of T lymphocytes (CD3+) and histiocytic cells (CD68+). Concentric fibromuscular intimal thickening reduced the vessel lumen, with similar changes in the arterioles. Exuberant fibrosis of the adjacent tissue was noted, accompanied by a dense inflammatory infiltrate of mononuclear cells, suggesting chronic fibrosing inflammation. No giant cells, granulomas, necrosis, or microorganisms were identified (*Figure 3*).

After discussion with rheumatology and pathology, the findings of non-infectious, non-necrotizing vasculitis with dense fibrosis, predominantly involving large vessels in a young adult with normal lipid profile, no drug history, and elevated CRP and ESR supported a presumptive diagnosis of TA with coronary involvement.

Glucocorticoids were initiated on the tenth post-operative day, with high-dose ‘pulse’ methylprednisolone (1 g/day) for 3 days, followed by 1 mg/kg/day of oral prednisolone. Prophylaxis against *Pneumocystis jirovecii* and prevention of corticosteroid-induced osteoporosis was started. A glucocorticoid-sparing agent was needed; however, two inconclusive interferon-gamma release assay tests, imposed a 30-day course of prophylactic pyridoxine and isoniazid first. Adalimumab (a monoclonal anti-tumour necrosis factor-alpha antibody) was chosen.

Following the initiation of immunosuppressive therapy, CRP and ESR normalized. Considering the patient’s presentation as an aborted sudden cardiac death, the presence of scar on CMR, as well the patient’s young age and the possibility of TA progression in the future, it was decided to implant a subcutaneous implantable cardioverter-defibrillator (S-ICD). On the fiftieth day of hospitalization, with a daily prednisolone dose of 20 mg, S-ICD was successfully implanted.

He was discharged after 54 days with a scheduled adalimumab administration in one week.

## Discussion

This case highlights several challenges. Coronary artery disease is an infrequent cause of sudden cardiac arrest (SCA) in those aged under 35, which led to a decision of postponing coronary angiography until echocardiogram and troponin values became available.

The optimal management of TA with coronary involvement and revascularization timing remains undefined.^7^ At the time of decision-making, the diagnosis of TA was not yet confirmed and the patient was a young adult with diffuse disease in whom recurrent ischaemia and ventricular arrhythmias were likely, which led the team to favour urgent revascularization. In patients with TA requiring revascularization, surgical revascularization is generally preferred over percutaneous intervention due to better long-term patency and lower rates of restenosis, particularly in diffuse or multi-vessel as in this patient disease.^8^ Diffuse arterial involvement makes graft selection and anastomosis sites critical, and while saphenous vein grafts are usually preferred,^7^ the LIMA was used in this case because the diagnosis was unknown at the time. A skeletonized harvesting technique was employed to minimize the risk of poor wound healing, and the patient did not experience any post-operative complications.

The differential diagnosis in a young patient with diffuse coronary artery disease includes non-atherosclerotic causes such as coronary artery dissection, congenital anomalies, and systemic inflammatory processes like vasculitis. A non-atherosclerotic inflammatory aetiology was first suspected after coronary angiography revealed diffuse, irregular lesions. This suspicion was reinforced intra-operatively, but histological analysis was critical for diagnosis, revealing mixed inflammatory infiltrates with concentric intimal thickening, lumen reduction, and disruption of the elastic lamina, pointing to elastic arteries vasculitis. In young adults, TA is the most common cause of such findings, even in the absence of giant cells. Exuberant adventitial and intimal inflammation, with luminal thickening and adherence to neighbouring structures, is characteristic of TA.^9^ Computed tomography angiography confirmed multi-arterial involvement.

Treatment involves corticosteroids to control inflammation, with high-dose IV for severe cases like this. To reduce relapse and steroid toxicity, non-glucocorticoid immunosuppressive agents are used. While methotrexate and azathioprine are commonly used, less myelosuppressive biologic agents such as anti-tumour necrosis factor agents (adalimumab), or anti-interleukin-6 agents (tocilizumab), have gained prominence.^10^ Adalimumab was chosen, given its expert’s endorsement, availability, clinical experience, and ability to allow disease monitoring via CRP.^11^

The patient remained hospitalized for 54 days due to the time required for histopathological confirmation of TA and to allow a gradual taper of glucocorticoids, which was necessary to safely proceed with S-ICD implantation while minimizing the risk of wound healing complications associated with high-dose steroids.

An S-ICD may seem unjustified give the reversible cause and partial recovery of left ventricular function. However, vasculitis has unpredictable progression and, in a young active patient, carries risks of exercised-induced ischaemia, as well as, ventricular arrhythmias due to the underlying pro-arrhythmogenic scar.

Finally, our case is also exceptional for the extensive and diffuse coronary artery vasculitis process, which distinguishes it from previously reported cases, in which either an ostial or focal pattern of coronary lesions were described.^4–7^

## Conclusions

This case emphasizes the importance of ruling out vasculitis in young patients with coronary artery disease. In TA, SCA as the first clinical manifestation, although unexpected, can occur. As in many systemic diseases with cardiac involvement, a multidisciplinary approach is essential in achieving a favourable outcome.

## Supplementary Material

ytag207_Supplementary_Data

## Data Availability

Non-identifiable data underlying this article will be made available upon reasonable request to the corresponding author.

## Lead author biography

Dr Débora da S. Correia is a third-year cardiology resident at Hospital de Santa Cruz, Lisbon. A graduate of the University of Lisbon, she is dedicated to advancing cardiovascular health through both clinical practice and research.

## Notes

**Consent:** The authors confirm that consent for submission and publication of this case report including images and text was obtained from the patient in line with COPE guidance.
